# Haematological alterations in malaria-infected refugees in South Africa

**DOI:** 10.1186/1475-2875-13-S1-P88

**Published:** 2014-09-22

**Authors:** Joyce Tsoka-Gwegweni, Uchenna Okafor

**Affiliations:** 1 University of KwaZulu-Natal, Durban, KwaZulu-Natal, South Africa

## Background

Though malaria in South Africa has been reduced dramatically in endemic areas, little is known about haematological changes associated with malaria infection among refugee populations that live in the cities. Aim The aim of the study was to describe haematological alterations among malaria-infected refugees in South Africa.

## Materials and methods

A cross-sectional study was conducted in 2013 at a refugee centre located in Durban, South Africa. The blood samples of 102 adult African refugees were screened for presence of malaria parasites using rapid diagnostic tests and microscopy. Haematological profiles were compared with standard normal values. The haematological alterations of both malaria infected and non-malaria infected participants were also compared.

## Results

Amongst the 102 participants, malaria infection was detected in 15.7% participants. The mean absolute haemoglobin (Hb) level was reduced in 16.3% of the malaria positive patients (9.2 g/dl) with an extremely low packed cell volume (PCV) of 28.3%. All other haematological parameters were similar for malaria infected and non-infected although slightly elevated in the former group.

## Conclusions

Anaemia was more common among participants with malaria infections than among non-infected participants. Other haematological alterations were detected in both malaria infected and uninfected participants, suggesting that infections other than malaria may be present. More research on a large sample is needed and should cover other areas and infections.

